# LncRNA TONSL-AS1 regulates miR-490-3p/CDK1 to affect ovarian epithelial carcinoma cell proliferation

**DOI:** 10.1186/s13048-020-00657-0

**Published:** 2020-05-15

**Authors:** Yan Liu, Ling Li, Xiangyang Wang, Ping Wang, Zhongxian Wang

**Affiliations:** 1 Department of Obstetrics and Gynecology, Wuhan No.1 Hospital, No. 215 Zhongshan Avenue, Wuhan City, Hubei Province 430022 PR China; 2 Department of Gastrointestinal Surgery, the Central Hospital of Wuhan, Tongji Medical College, Huazhong University of Science and Technology, Wuhan City, Hubei Province 430014 PR China

**Keywords:** Ovarian epithelial carcinoma, TONSL-AS1, Survival, miR-490-3p, CDK1

## Abstract

**Background:**

LncRNA TONSL-AS1 has been characterized as a critical player in gastric cancer. By analyze the TCGA dataset, we observed the upregulation of TONSL-AS1 in ovarian epithelial carcinoma (EOC). We therefore investigated the involvement of TONSL-AS1 in EOC.

**Methods:**

The differential expression of TONSL-AS1 in EOC was first explored by analyzing the TCGA dataset. The effects of overexpression of TONSL-AS1 and miR-490-3p on the expression of CDK1 mRNA and protein in OVCAR3 cells were evaluated by qPCR and western blot, respectively. CCK-8 assay was performed to investigate the effects of overexpression of TONSL-AS1, miR-490-3p and CDK1 on proliferation of OVCAR3 cells.

**Results:**

We observed that TONSL-AS1 was upregulated in EOC tumor tissues from EOC patients, and its high expression level was correlated with poor survival. Dual luciferase assay and RNA interaction prediction showed the direct interaction between TONSL-AS1 and miR-490-3p. However, overexpression of miR-490-3p did not affect the expression of TONSL-AS1. Instead, overexpression of TONSL-AS1 resulted in the upregulation of CDK1, a target of miR-490-3p, in EOC cells. Overexpression of TONSL-AS1 and CDK1 resulted in increased proliferation rate of EOC cells. Overexpression of miR-490-3p played an opposite role and reduced the effects of overexpression of TONSL -AS1 and CDK1.

**Conclusions:**

Therefore, TONSL-AS1 may regulate miR-490-3p/CDK1 to affect EOC cell proliferation.

## Background

Ovarian carcinoma is the 7th most commonly diagnosed malignancy among females worldwide and the 10th most common cancer in China [1]. In 2018, ovarian cancer caused 184,799 deaths, accounting for 1.9% of all cancer deaths. In the same year, a total of 295,414 new cases of ovarian cancer were diagnosed [2]. More than 85% of ovarian carcinomas are ovarian epithelial carcinoma (EOC) [3]. With the development of cancer therapies, such as local and systemic chemotherapy, radiation therapies and surgical resection of primary tumors, the overall survival of EOC has been improved significantly during the past several decades [4, 5]. However, prognosis of EOC patients diagnosed at advanced stages is still poor [6]. Therefore, novel therapies are of great importance.

It has been well established that genetic factors are critical players in the pathogenesis of EOC [7]. Identification of critical players in EOC may provide new targets for the development of targeted therapies [8]. Cyclin-dependent kinase 1 (CDK1) is a type of threonine/serine kinase that has critical roles in cell cycle regulation [9]. In cancer biology, CDK1 is overexpressed and the inhibition of CDK1 is considered as a potential therapeutic approach for cancer treatment [10]. In effect, some tumor suppressive miRNAs, such as miR-490-3p, can inhibit EOC progression by targeting CDK1 [11]. It is known that the functions of miR-490-3p in cancer biology can be mediated by certain lncRNAs [12]. TONSL-AS1 (on chromosome 8) is a recently identified tumor suppressive lncRNA in gastric cancer [13]. In gastric cancer, TONSL-AS1 activates TONSL to suppress cancer cell proliferation [13]. Through the analysis of TCGA dataset we observed that TONSL-AS1 was also upregulated in EOC (more than 3 times increase). In addition, it is predicted that TONSL-AS1 can interact with miR-490-3p. We therefore investigated the interactions between TONSL-AS1 and miR-490-3p in EOC.

## Methods

### Study subjects and specimens

A total of 62 pairs of EOC and adjacent (within 2 cm around tumors) non-tumor tissue samples were obtained from 62 EOC cancer patients (all females, age range 45 to 67 years old, mean age 54.8 ± 4.7 years old) who underwent the diagnosis of EOC through MRI-guided biopsy during March 2015 and March 2017. Inclusion criteria: 1) patients diagnosed for the first time; 2) no systemic or local treatments were initiated. Exclusion criteria: 1) recurrence after therapies; 2) patients complicated with other severe clinical disorders. All tissue samples were immediately frozen in liquid nitrogen and stored at − 80 °C before use. All patients were informed of the experiments included in this project and signed the informed consent.

### OEC cell line

OVCAR3 OEC cell line (ATCC, USA) was used in this study. Cell culture medium was composed of 10% FBS and RPMI 1640 media (100 μg/mL streptomycin and 100 U/mL penicillin). Cell culture conditions were 37 °C, 5% CO_2_ and 95% humidity.

### Therapies and follow-up

The determination of therapies is mainly made based on clinical stages and patients’ health conditions. Targeted therapies, surgeries, chemotherapy, radiation therapy or their combinations were performed. From the day of admission, a 5-year follow-up was performed through outpatient visit or telephone in a monthly manner to monitor the survival conditions of the 62 patients. All patients completed the follow-up or died of EOC during follow-up.

### Transient transfections

Full length cDNA of TONSL-AS1 (Accession: NR_109770.1; 1196 nt) and CDK1 (Accession: NM_001786.5; 1889 nt) were inserted into pcDNA 3.1 vector (Invitrogen) to establish expression vectors. Mimic of miR-490-3p and miRNA NC (negative control) were synthesized by Invitrogen. Cells were harvested at confluence of 70–80%, followed by the transfection of 40 nM miRNA (miRNA NC as NC group) or 10 nM vector (empty vector was NC group) into 10^6^ cells. Cells were harvested at 24 h post-transfection to perform the following experiments. Control cells were untransfected cells in all cases.

### Dual-luciferase reporter assay

TONSL-AS1 cDNA was inserted into pmirGLO Dual-luciferase miRNA Target Expression Vector (Promega, USA). TONSL-AS1 luciferase vector combined with miR-490-3p (miR-490-3p group) or NC miRNA was transfected into cells through the same methods aforementioned. Dual-Luciferase Reporter Assay System (Promega) was used to measure luciferase activity.

### RNA and qPCR analysis

TRIZOL reagent (Invitrogen) was used to extract total RNAs. With 85% ethanol used in RNA precipitation and washing, miRNAs were retained. Genomic DNAs were removed by incubating RNA samples with DNA eraser (Takara, Japan) at 37 °C for 2 h. First strand cDNA synthesis kit (Takara) was used for reverse transcriptions (RTs) with poly (T) as primer and RNA samples as template. With cDNA at template, qPCR mixtures were prepared using SYBR Green dye (Takara). Expression levels of TONSL-AS1 and CDK1 mRNA were normalized with GAPDH as endogenous control. Measurement of the expression levels of miR-490-3p was performed using mirVana qRT-PCR miRNA Detection Kit (Thermo Fisher Scientific) with all operations performed following the manufacturer’s instructions. Fold changes of gene expression were normalized using 2^-ΔΔCt^ method.

### Western blotting

RIPA buffer (Sigma-Aldrich) was used to lysis cells on ice with the existence of phosphatase and proteinase inhibitors (Sigma-Aldrich). Under 12,000x g, cell lysates were centrifuge at 4 °C for 15 min. BCA assay as performed to measure protein concentrations, followed by denaturation in boiling water for 10 min. Electrophoresis was performed to separate different proteins and gel transfer was performed onto PVDF membrane. Membranes were blocked in 5% BSA (TBST), followed by incubation with CDK1 (ab131450, Abcam) and GAPDH (ab9485, Abcam) primary antibodies at 4 °C for 18 h, followed by incubation with secondary antibody of HRP Goat Anti-Rabbit (IgG) (ab6721, Abcam). Signal productions were performed using ECL™ Blocking Agent GE Healthcare (RPN2125, Sigma-Aldrich). Image J v1.46 software was used for data normalizations.

### CCK-8 assay

Transfected (NC and experimental groups) and untransfected (C group) cells were counted and seeded to a 96-well plate (4000 cells/well) for adhesion. Cells were cultivated under aforementioned conditions and each well was added with CCK-8 solution (10 μl, Dojindo Molecular Technologies, USA) at 4 h before the collection of cells. Cells were collected at 24, 48, 72 and 96 h post-transfection. Then, a Microplate Reader (Bio-Rad, USA) was used to measure OD values at 450 nm.

### Statistical analysis

Three independent biological replicates were included in each assay. Mean values were calculated and used for data comparisons. Paired Student’s t-test was used for comparisons between EOC and non-tumor tissues. For comparisons among multiple groups, ANOVA (one-way) combined with Tukey test was used. Patients were divided into high and low TONSL-AS1 level groups (*n* = 31) with the mean level of expression in TONSL-AS1 as cutoff value. Kaplan-Meier survival plotter was used to plot survival curves, and log-rank test was used to compare curves. *P* < 0.05 was considered as statistically significant.

## Results

### Upregulation of TONSL-AS1 is a potential prognostic factor for EOC

**Fig. 1:**
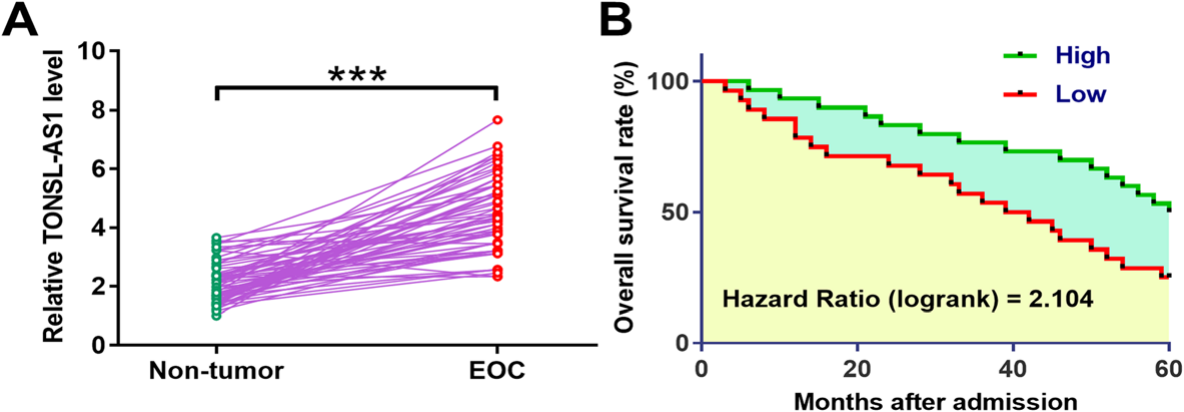
Upregulation of TONSL-AS1 is a potential prognostic factor for EOC. Expression levels of TONSL-AS1 in EOC and non-tumor tissues from the 62 EOC patients included in this study by performing qPCR (**a**). Patients were divided into high and low TONSL-AS1 level groups (*n* = 31) with the mean level of expression in TONSL-AS1 as cutoff value. Kaplan-Meier survival plotter was used to plot survival curves, and log-rank test was used to compare curves (**b**). PCR reactions were repeated 3 times and data were expressed as mean values, ***, *p* < 0.0001

The differential expression of TONSL-AS1 in EOC was first explored by analyzing the TCGA dataset. It was observed that the expression levels of TONSL-AS1 were significantly higher in EOC tissues than that in non-tumor tissues (1.34 vs. 0.37). To further confirm its upregulation, expression levels of TONSL-AS1 in EOC and non-tumor tissues from the 62 EOC patients included in this study were determined by qPCR. Compared to non-tumor tissues, the expression levels of TONSL-AS1 were significantly higher in EOC tissues (Fig. 1a, *p* < 0.0001). Survival curves were plotted and compared through aforementioned methods. Compared to patients in low TONSL-AS1 level group, patients in high TONSL-AS1 level group experiences significantly lower overall survival rate (Fig. 1b). These data suggested that upregulation of TONSL-AS1 may participate in EOC and predict the poor survival of EOC patients.

### MiR-490-3p interacted with TONSL-AS1 but did not regulate its expression

**Fig. 2:**
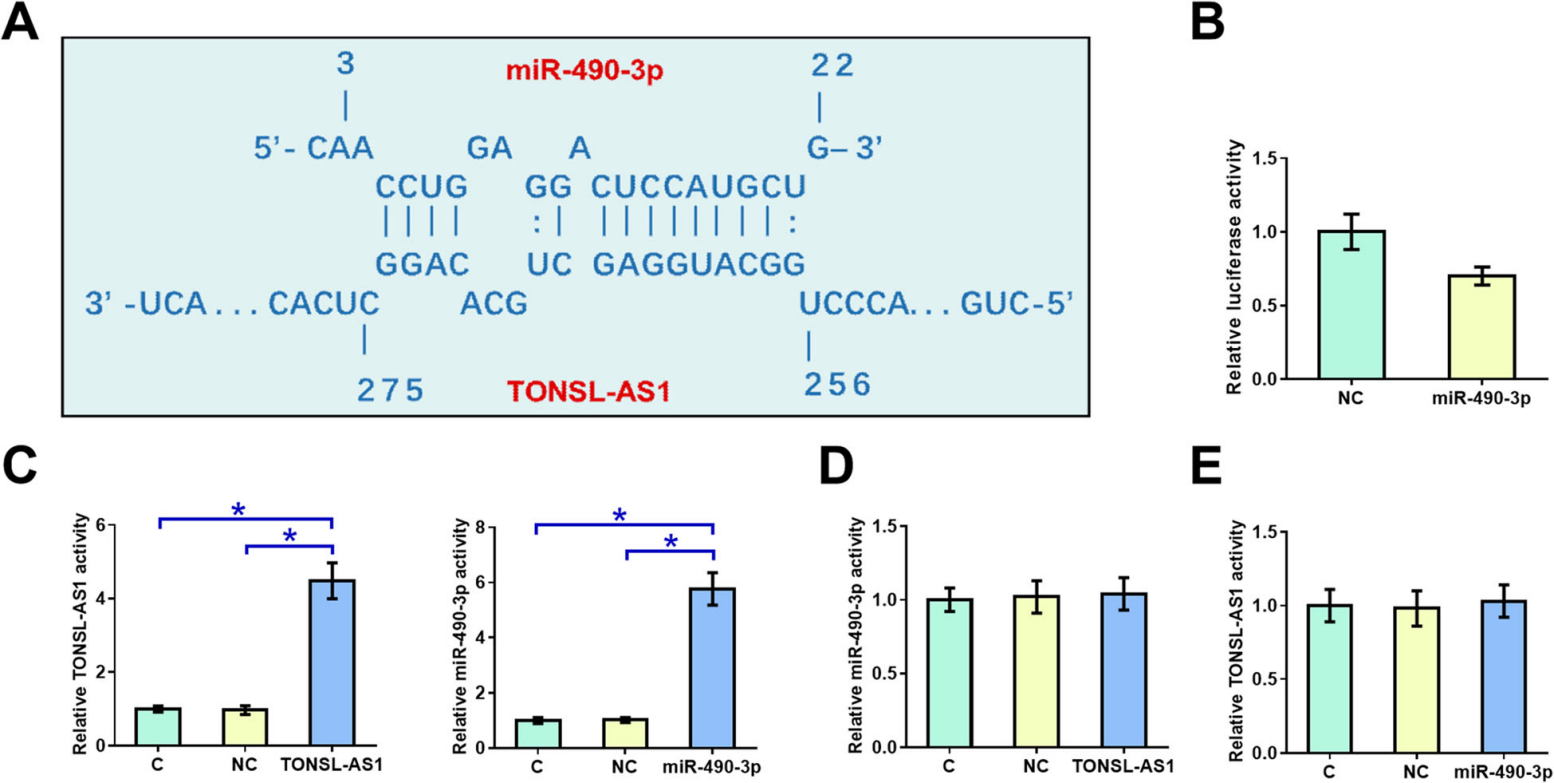
MiR-490-3p interacted with TONSL-AS1 but did not regulate its expression. RNA interaction prediction performed using IntaRNA (http://rna.informatik.uni-freiburg.de/IntaRNA/Input.jsp) showed that miR-490-3p can bind TONSL-AS1 (**a**). Dual luciferase reporter assay was performed by transfecting TONSL-AS1 + miRNA NC (NC group) or TONSL-AS1 + miR-490-3p (miR-490-3p) into OVCAR3 cells (**b**). To analyze the relationship between them, OVCAR3 cells were transfected with TONSL-AS1 expression and miR-490-3p mimic. Overexpression of TONSL-AS1 and miR-490-3p was confirmed by qPCR (**c**). The effects of TONSL-AS1 overexpression on miR-490-3p (**d**) and the effects of overexpression of miR-490-3p on TONSL-AS1 (**e**) were also analyzed by qPCR. Experiments were repeated 3 times and data were expressed as mean values, *, *p* < 0.05

RNA interaction prediction performed using IntaRNA (http://rna.informatik.uni-freiburg.de/IntaRNA/Input.jsp) showed that miR-490-3p can bind to TONSL-AS1 (Fig. 2a). Dual luciferase reporter assay was performed to analyze the interaction between them. Compared to OVCAR3 cells transfected with TONSL-AS1 + miRNA NC (NC group), cells transfected with TONSL-AS1 + miR-490-3p (miR-490-3p) showed significantly reduced relative luciferase (Fig. 2b, *p* < 0.05). To analyze the relationship between them, OVCAR3 cells were transfected with TONSL-AS1 expression and miR-490-3p mimic. Overexpression of TONSL-AS1 and miR-490-3p was confirmed by qPCR at 24 h post-transfection (Fig. 2c, *p* < 0.05). Compared to NC and C groups, overexpression of TONSL-AS1 did not affect the expression of miR-490-3p (Fig. 2d), and overexpression of miR-490-3p also did not affect the expression of TONSL-AS1 (Fig. 2e). Therefore, TONSL-AS1 can interact with miR-490-3p, while TONSL-AS1 is unlikely a target of miR-490-3p.

### Upregulation of CDK1 was observed after the overexpression of TONSL-AS1

**Fig. 3:**
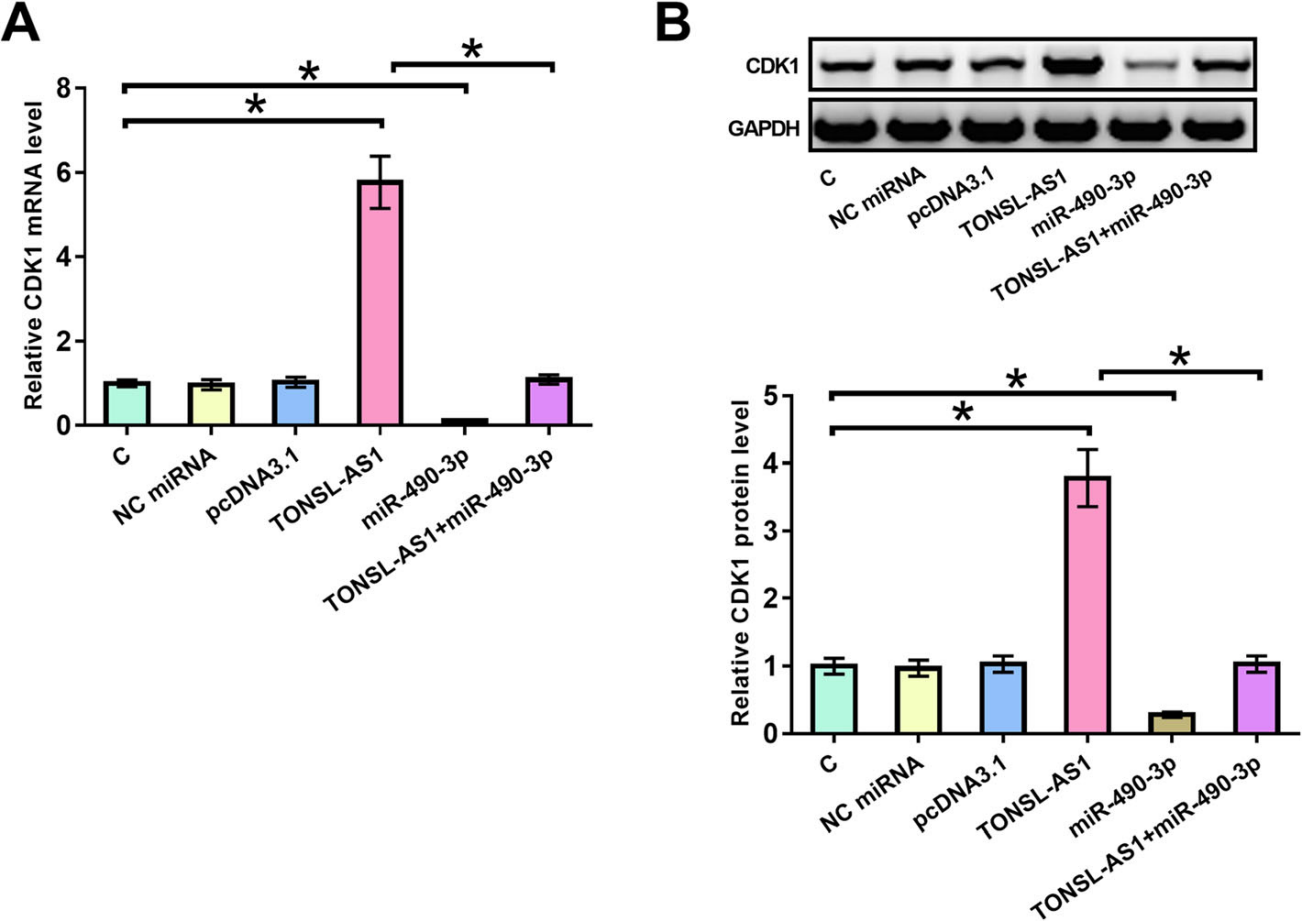
Upregulation of CDK1 was observed after the overexpression of TONSL-AS1. The effects of overexpression of TONSL-AS1 and miR-490-3p on the expression of CDK1 mRNA (Fig. 3**a**) and protein (Fig. 3**b**) in OVCAR3 cells were analyzed by qPCR and western blot, respectively. Experiments were repeated 3 times and data were expressed as mean values, *, *p* < 0.05

CDK1 is a target of miR-490-3p [11]. To explore the possibility that TONSL-AS1 may sponge miR-490-3p, the effects of TONSL-AS1 and miR-490-3p overexpression on the expression of CDK1 mRNA (Fig. 3a) and protein (Fig. 3b) in OVCAR3 cells were analyzed by qPCR and western blot, respectively. Compared to untransfected cells (C) or cells transfected with miRNA mimic or empty pcDNA3.1 vector, TONSL-AS1 overexpression resulted in significant upregulation of CDK1, a target of miR-490-3p (*p* < 0.05). In contrast, miR-490-3p overexpression resulted in downregulation of CDK1 and reduced effects of TONSL-AS1 overexpression (*p* < 0.05). Therefore, TONSL-AS1 may sponge miR-490-3p to upregulate CDK1.

### TONSL-AS1 regulated miR-490-3p/CDK1 axis to promote cell proliferation

**Fig. 4:**
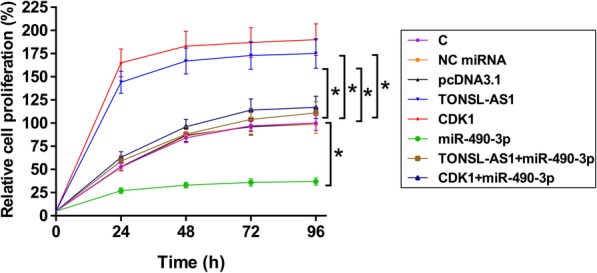
TONSL-AS1 regulated the miR-490-3p/CDK1 axis to promote cell proliferation. CCK-8 assay was performed to analyze the effects of overexpression of TONSL-AS1, miR-490-3p and CDK1 on proliferation of OVCAR3 cells. Experiments were repeated 3 times and data were expressed as mean values, *, *p* < 0.05

CCK-8 assay was performed to analyze the effects of overexpression of TONSL-AS1, miR-490-3p and CDK1 on proliferation of OVCAR3 cells. Compared to untransfected cells (C) or cells transfected with miRNA mimic or empty pcDNA3.1 vector, overexpression of TONSL-AS1 and CDK1 resulted in increased proliferation rate of EOC cells. Overexpression of miR-490-3p played an opposite role and reduced the effects of overexpression of TONSL-AS1 and CDK1 (Fig. 4, *p* < 0.05). Therefore, overexpression of TONSL-AS1 promoted EOC cell proliferation possibly by sponging miR-490-3p to upregulate CDK1.

## Discussion

This study investigated the roles of TONSL-AS1 in EOC. We found that TONSL-AS1 was upregulated in EOC and was correlated with the survival of EOC patients. In addition, TONSL-AS1 may regulate the miR-490-3p/CDK1 axis to promote the proliferation of EOC cells.

A recent study reported the downregulation of TONSL-AS1 in gastric cancer and its tumor suppressive roles in this disease [13]. It was observed that overexpression of TONSL-AS1 inhibited the in vitro cell proliferation, invasion and migration, and also suppressed in vivo cell tumorigenesis [13]. Consistently, by analyzing TCGA dataset we also observed the downregulation of TONSL-AS1 in gastric cancer (0.68 vs. 0.64, STAD). However, Wang et al. reported about 2-fold differences in expression levels of TONSL-AS1 between gastric cancer and non-tumor tissues [13]. Therefore, further studies may be required to include more patients to further confirm the downregulation of TONSL-AS1 in gastric cancer.

The upregulation of TONSL-AS1 in EOC was further confirmed by measuring its expression levels in both EOC and non-tumor tissues derived from the 62 EOC patients included in this study. It is known that the same lncRNA may play opposite roles in different types of cancer [14, 15]. For instance, lncRNA TUG1 suppress glioma by promotes osteosarcoma [14, 15]. In this study, we observed the oncogenic roles in EOC by promoting the proliferation of cancer cells. Therefore, TONSL-AS1 may play different roles in different cancers. By analyzing the TCGA dataset we also observed the different expression patterns of TONSL-AS1 in different types of cancer.

MiR-490-3p is a well-characterized tumor suppressive miRNA with downregulated expression pattern in different types of cancer [16, 17]. A recent study showed that miR-490-3p suppressed EOC progression by targeting CDK1. Our study confirmed the targeting of CDK1 by miR-490-3p in EOC cells by performing overexpression experiments. Our data support the idea that TONSL-AS1 may sponge miR-490-3p to upregulate CDK1 due to the observation that: 1) miR-490-3p can interact with TONSL-AS1 but did not affect its expression; 2) Overexpression of TONSL-AS1 resulted in upregulation of CDK1, the downstream target of miR-490-3p.

In conclusion, TONSL-AS1 is upregulated in EOC and may sponge miR-490-3p to upregulate CDK1, thereby promoting cancer cell proliferation.

## Data Availability

The analyzed data sets generated during the study are available from the corresponding author on reasonable request.

## Abbreviation

EOC: Ovarian epithelial carcinoma
